# Abdominal Wall Clear Cell Carcinoma: Case Report of a Rare Event with Potential Diagnostic Difficulties

**DOI:** 10.1155/2019/1695734

**Published:** 2019-07-18

**Authors:** Maria del Mar Rivera Rolon, Dyron Allen, Gwyn Richardson, Cecilia Clement

**Affiliations:** University of Texas Medical Branch, USA

## Abstract

Clear cell carcinoma (CCC) is a well-known aggressive histological type of carcinoma, predominantly seen in ovary and endometrium. However, CCC arising in abdominal wall is a very rare event. We report a case of a 48-year-old woman with an abdominal wall mass at her cesarean section (c-section) scar, which increased in size and became painful in the last months. Radiology revealed a 7 cm mass in the right inferior rectus muscle sheath, suggestive of endometriosis. An irregular, firm mass was resected, densely adherent to the rectus muscle and pubic bone. Frozen section revealed a multicystic lesion with minimal cytologic atypia, and a benign cystic neoplasm was favored. However, permanent sections showed marked nuclear atypia, hobnail morphology, and areas of infiltrative growth within fibrous stroma. No benign endometrial glands were found, although fibrosis and hemorrhage were present. Napsin-A, racemase, and PAX-8 were positive, consistent with CCC, likely arising within a c-section endometriosis focus. Although CCC usually presents with moderate to marked nuclear atypia, it can be mild and, especially in cases with a predominant cystic pattern, create diagnostic difficulties. An endometriosis-associated malignancy should be considered in the differential with any enlarging nodule or increasing pain within an abdominal wall scar.

## 1. Introduction

Clear cell carcinoma (CCC) of the female genital tract can arise in the ovary, endometrium, and cervicovaginal region, as well as in peritoneal and other extrapelvic sites. However, CCC arising within an abdominal wall scar is rare, and when it is found, it is most commonly related to underlying endometriosis. Although the pathophysiology is controversial, it usually affects women that underwent operations such as hysterectomy or c-section, with a probable explanation for this occurrence being malignant transformation of an endometriosis focus within the abdominal scar [1]. Malignant transformation has been reported in approximately 1% of the endometriosis cases, and most frequently this transformation takes place at the ovary, whereas its occurrence in an abdominal wall scar is rare [2–4].

Here we report a case of CCC arising from an abdominal wall c-section scar, with a discussion of diagnostic criteria and potential diagnostic difficulties. We also present a review of the available literature relative to this unusual event.

## 2. Case Presentation

### 2.1. Clinical Information

This is a 48-year-old woman with no previous cancer history, who presented with complaint of pain at the inferior edge of her midline c-section scar. Surgical and obstetric histories were significant for three c-sections, excision of endometriosis from c-section scar (20 years ago), and hysterectomy and bilateral salpingo-oophorectomy for leiomyomata and endometriosis of left fallopian tube (4 years ago). No positive family history of cancer was reported. Since her hysterectomy, the patient reported decreased sex drive, dyspareunia, weight gain, vaginal dryness, and abdominal pain. She was taking oral estradiol but admits being noncompliant. Eventually, she was switched to vaginal estradiol (10 mcg). No cancer markers were performed. The patient underwent imaging analysis and surgical excision of the mass as described below. She was scheduled for adjuvant chemotherapy with Paclitaxel and Carboplatin; however, she has been lost to follow-up.

### 2.2. Imaging Analysis

**Figure 1:**
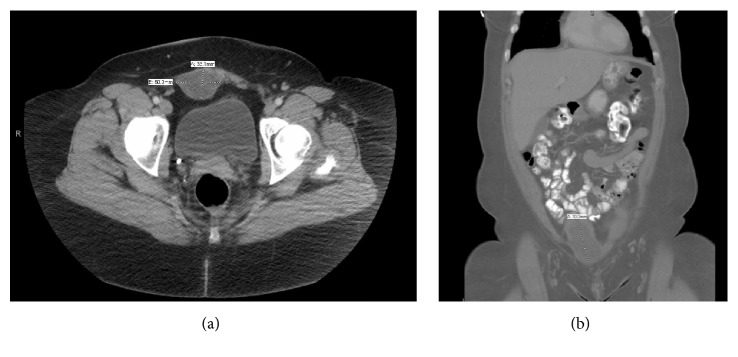
CT image of the abdominal mass shows a right lower rectus muscle heterogeneous collection: axial (a) and coronal (b) views.

Computed tomography scan (CT-scan) revealed a 7.0 × 5.0 × 3.3 cm heterogeneous collection at the right inferior rectus sheath and concluded as most likely a scar endometriosis (Figure 1). The patient reported awareness of the mass for years, but in the last months she noticed it increased in size and became painful.

A follow-up PET imaging done after surgery revealed residual uptake in the anterior right abdominal wall, most likely postsurgical effect; however, residual disease could not be completely excluded.

### 2.3. Gross Description

The patient underwent exploratory laparotomy, and an irregular, cystic, firm mass was identified, located deep to the rectus muscle in the midline. Dense adhesions and scarring were noted between the mass, rectus muscles, and pubic bone. The mass was excised and submitted for intraoperative consultation. The specimen was received fragmented, consisting of four irregular shaped soft tissue fragments, the largest measuring 5.0 × 3.0 × 2.0 cm and the smallest 2.0 × 0.7 × 1.2 cm. This tissue was firm and hemorrhagic, composed of similarly sized, microcystic spaces. The other pieces consisted of fibroadipose tissue.

### 2.4. Histology Description

**Figure 2:**
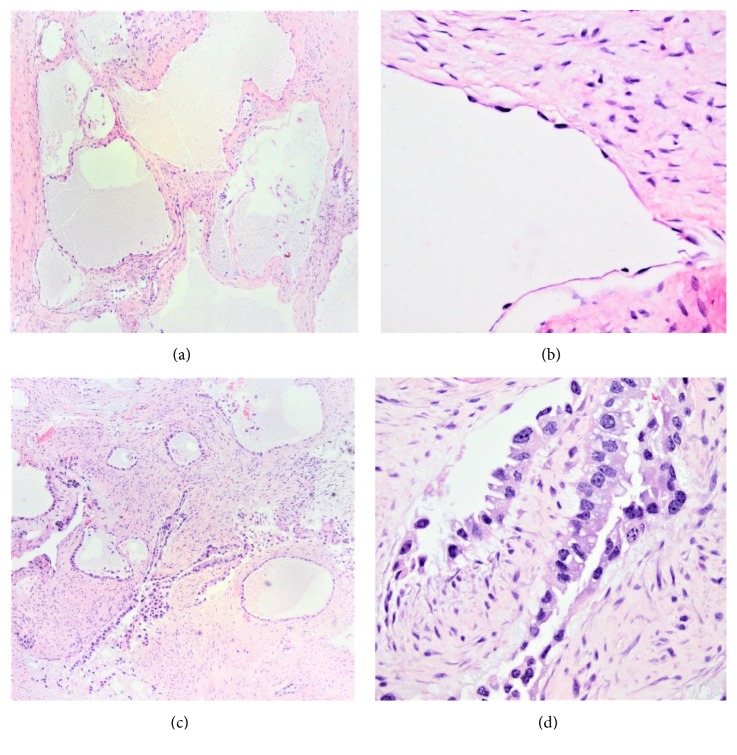
Histologic images of the tumor. (a) Multicystic bland appearance of the tumor, as seen on frozen section (H&E stain, X40). (b) Higher magnification of cystic spaces lined by bland-appearing, flattened cells (H&E stain, X200). (c) In other areas the tumor shows irregular infiltrating glands with fibrous stroma. (d) Highly atypical cells, with hobnail morphology and prominent nucleoli, lining the infiltrating tumor (H&E stain, X400).

Microscopic examination of one representative section submitted for frozen section showed a multicystic lesion with large cystic spaces lined by a single layer of flattened to cuboidal epithelium with bland hyperchromatic nuclei and occasional enlarged nuclei (Figures 2(a) and 2(b)). A “benign multi-cystic neoplasm of undetermined origin” was favored, with final diagnosis deferred to permanent sections evaluation.

The specimen was entirely submitted for permanent sections. Additional sections demonstrated again a predominantly cystic pattern and some tubules, lined by highly atypical cells admixed with more bland-appearing ones, and areas of infiltrative growth within a fibrous desmoplastic stroma. The malignant cells showed clear to eosinophilic cytoplasm, enlarged, hyperchromatic nuclei, prominent nucleoli, and hobnail morphology in areas (Figures 2(c) and 2(d)). Mitotic activity was increased. No other growth patterns characteristic of CCC such as papillary areas with hyalinized stromal cores or solid areas or hyaline globules were identified. After a thorough search, no benign endometrial glands were found, although extensive fibrosis likely representing scarring, associated with areas of hemorrhage, was present. Inked edges of the specimen appeared positive for malignancy; however, margins status could not be accurately assessed given the fragmentation of the specimen.

**Figure 3:**
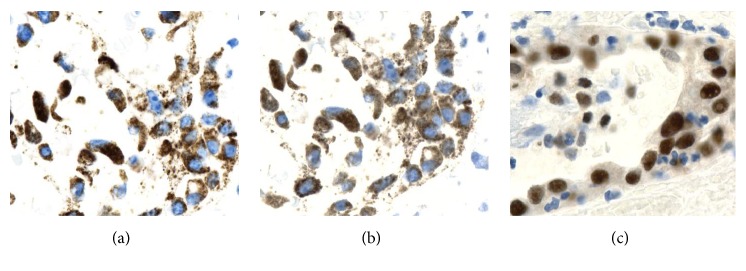
Immunohistochemical profile. Tumor cells are immunoreactive for AMACR, cytoplasmic staining (a), Napsin-A, cytoplasmic staining (b), and PAX-8, nuclear staining (c) (X400).

Immunohistochemical stains were performed (Figure 3). Neoplastic cells showed strong immunoreactivity for AE1/AE3, napsin A, racemase, and PAX-8; p53 was weak positive (wild type) and p16 was patchy. On the other hand, tumor cells were negative for calretinin, D2-40, CK 5/6, CA-IX, Melan-A, ER, and WT-1. Hence, the morphologic findings and the immunohistochemical profile were consistent with the diagnosis of clear cell carcinoma arising in an abdominal wall scar.

## 3. Discussion

Scar endometriosis has been known at least since 1942 [5]. Although endometriosis implants are typically observed after c-section (0.03% to 1%) or hysterectomy, they have also been reported to be associated with episiotomy, trocar scars, appendectomy, and hernia repair scars [6–8]. Malignant transformation of endometriosis associated with surgical scars is very rare, with an estimated incidence of no more than 1% [2–4, 9, 10]. CCC is the histological subtype more often present in these cases, followed by endometrioid adenocarcinoma [8]. Several risk factors have been described for malignant extraovarian endometriosis: hyperestrogenism, carcinogens and cocarcinogens (such as dioxin and polychlorinated biphenyls), and some genetic anomalies (loss of heterozygosity on chromosome 5q) [11, 12].

Microscopically, CCC can have a variety of architectural patterns within the same tumor, including cystic, tubulocystic, solid, and papillary growth with characteristic hyalinized fibrovascular cores [13]. CCC is usually recognized histologically by cuboidal to polygonal cells with moderate to abundant clear cytoplasm, although cytoplasm can be eosinophilic or granular in some cases. Centrally located nuclei show pleomorphism and hyperchromasia with prominent nucleoli. Characteristic hobnail cells are often encountered, with globular nuclei that bulge into the lumen of cystic spaces. In general, this tumor exhibits high-grade nuclear features, although a spectrum of nuclear atypia may also be present within the same tumor, and it can lead to misdiagnosis [13, 14].

The clinical differential diagnosis of palpable abdominal wall masses includes benign entities such as hernia, hematoma, abscess, desmoid tumors, and other soft tissue neoplasms. If a malignant neoplasm is found in a patient with a history of gynecologic or obstetric surgery, the possibility of a primary malignancy arising from endometriosis should be considered, although cutaneous metastases from a Mullerian or nongynecological primary should be ruled out [4].

Tumors with a predominant cystic or tubulocystic pattern, lined by flat to cuboidal tumor cells with mild nuclear atypia, may cause diagnostic difficulties, in particular with small biopsies and frozen sections [13, 14]. During frozen section evaluation in our case, the bland appearance of the tumor cells in a cystic pattern was misleading. A benign mesothelial neoplasm such as adenomatoid tumor, or a low-grade serous neoplasm, was initially considered in the differential. However, the classical features of low-grade serous tumors, such as bud-like or papillary growth, and psammoma bodies were lacking. Similarly, characteristic features of adenomatoid tumor such as tubule-glandular structures, cytoplasmic bridges, or lymphoid aggregates were also lacking. In these situations, careful examination of multiple sections is required and highly recommended for this diagnosis. Although there is no standard number of sections to submit for this diagnosis in particular, as a general rule at our institution we consider one section per centimeter of maximal diameter of the tumor. It is important to grossly identify visually different zones of the specimen. However, if the specimen is small, then all the tissue should be submitted.

Proper diagnosis should also take advantage of immunohistochemical panels as needed. Immunostains can support a gynecological origin versus other tumors with clear cells, such as renal cell carcinoma (RCC) or adrenocortical carcinoma. Napsin A, racemase, and hepatocyte nuclear factor (HNF-1b) strong immunopositivity will support a diagnosis of CCC [15], whereas RCC is usually negative to these and reactive with CA-IX, RCC antigen, EMA, and CD10. Adrenocortical carcinoma expresses SF-1, Melan-A, calretinin, S100, and inhibin [4]. WT-1 is the most important marker to distinguish serous tumors from CCC, whereas calretinin, keratin 5/6, and WT-1 will be positive in mesothelial tumors, as opposed to CCC [2] (Table 1).

To the best of our knowledge, 29 cases of CCC arising in abdominal wall endometriosis have been reported in the literature between 1986 and 2017 (Table 2). The average age at diagnosis in these reports was 44 years. Virtually all cases had a history of previous c-section, with only three exceptions: one case followed myomectomy [24], one followed tubal ligation with oophorectomy [29], and one followed laparotomy for endometrioma [26]. As shown in Table 2, coexisting endometriosis implants were identified only in 20 cases (66.6%, 20/30), although seven cases, including the present case, had a previous history of excision of endometriotic nodules in the c- section scar [11, 22, 23, 27, 33]. In our case, additional sampling with an exhaustive search for a residual focus of endometrial glands was unsuccessful. However, gross inspection and microscopy revealed areas of tumor hemorrhage and fibrosis. These findings, in addition to the history of scar endometriosis excision, suggest a preexisting endometriotic origin. The absence of histologically assessed endometriosis foci could be interpreted as either a sampling problem or a consequence of the complete replacement of normal tissue due to massive neoplastic proliferation [2]. Zhao and collaborators have investigated the pathogenesis of ovarian CCC and proposed that endometriosis is the underlying precursor for CCC. About half of the ovarian CCCs in their study did not contain endometriosis in the ovarian tumor, and they postulate that tumor progression and overgrowth of the precursor elements appear to be the most likely explanation in those cases [37]. When diagnosing a malignancy from preexisting endometriosis, it is important to document the presence of benign endometrial tissue in the tumor. Therefore, examination of multiple sections is required and highly recommended for this diagnosis. Although not strictly necessary from a diagnostic standpoint, it can help to determine the true incidence and understanding the natural history of these carcinomas [8, 10].

The diagnosis of malignant transformation of abdominal wall endometriosis is still a challenge for clinicians. There are no characteristic symptoms or markers of malignant transformation, and imaging may only show endometrioma with fast growth. Similarly, imaging in our case suggested a scar endometriosis, and no malignant transformation was suspected before surgery. Given the increased rate of c-sections registered in the last years, we may expect a parallel increase of endometriosis implants in the c-section scar and, therefore, occurrence of CCC of the abdominal wall should be kept in mind [2].

## 4. Conclusion

CCC arising from abdominal wall is a very rare event. However, it should be considered in the differential diagnosis of any patient who presents with an enlarging abdominal wall mass or increasing pain within a surgical scar. Although CCC is a high-grade malignancy, the degree of atypia may vary; it can be mild and, especially in cases with a predominantly cystic pattern, can cause diagnostic difficulties.

## Figures and Tables

**Table 1:** Differential diagnosis of Mullerian clear cell carcinoma.

Diagnosis	Histologic features	Immunohistochemical markers
Mullerian clear cell carcinoma	Tubulocystic, papillary and solid patterns; small, frequently hyalinized papillae. Polygonal to cuboidal to flattened cells, with clear to eosinophilic cytoplasm; hobnail morphology.	Napsin-A, racemase, hepatocyte nuclear factor (HNF-1b)

High grade serous carcinoma	Branching papillary fronds, slit-like fenestrations, glandular complexity. Moderate to marked pleomorphism, prominent nucleoli, increased mitotic rate.	WT1, p53

Clear cell renal cell carcinoma	Tubulocystic or less commonly papillary pattern, cells with clear cytoplasm, distinct but delicate cell boundaries, small, thin walled, “chicken wire” vasculature.	CA-IX, RCC antigen, EMA, CD10

Mesothelioma	Epithelial or biphasic tumor with tubular, papillary or solid patterns. Tumor cells have moderate atypia and low mitotic rate.	Calretinin, CK5/6, WT-1, D2-40

Adrenocortical carcinoma	Different growth patterns; tumor cells with vacuolated to densely eosinophilic cytoplasm, usually marked nuclear atypia and increased mitotic rate.	SF-1, Melan-A, calretinin, S100, inhibin

**Table 2:** Clear cell carcinoma arising in abdominal wall endometriosis (n=30).

Author	Year reported	Age	Previous GYN Surgery	Coexisting endometriosis	Follow up (months)	Outcome
Schnieber & Wagner-Kolb [16]	1986	40	CS	Yes	18	DOD
Hitti et al [17]	1996	46	CS	Yes	30	NED
Miller et al [18]	1998	38	CS	Yes	60	NED
Park et al [19]	1999	54	CS	Yes	1.5	NED
Ishida et al [20]	2003	56	CS	No	48	DOD
Alberto et al [21]	2006	38	CS; TAH + BSO for pelvic endometriosis.	No	NA	NA
Sergent et al [22]	2006	45	CS; scar endometriotic nodules excisions.	Yes	6	DOD
Razzouk et al [23]	2007	46	CS; scar endometriotic nodules excisions.	Yes	6	DOD
Achach et al [24]	2008	49	Myomectomy	NA	18	Recurrence
Rust et al [25]	2008	42	CS; TAH	Yes	NA	NA
Bats et al [11]	2008	38	CS; scar endometriotic nodule excision.	Yes	4	Recurrence
Williams et al [1]	2009	53	CS	No	11	DOD
Matsuo et al [26]	2009	37	Laparotomy for endometrioma.	No	18	Recurrence
Bourdel et al [27]	2010	43	CS; scar endometriotic nodule excision.	Yes	22	DOD
Yan et al [28]	2011	41	CS; scar endometriotic nodules excisions.	No	24	NED
Shalin et al [4]	2012	47	CS	Yes	7	NED
Mert et al [29]	2012	42	Tubal ligation; oophorectomy.	Yes	26	NED
Mert et al [29]	2012	51	CS; TAH	Yes	49	NED
Sawazaki et al [30]	2012	41	CS	Yes	NA	NA
Li et al [31]	2012	49	CS	No	8	NED
Ijichi et al [9]	2014	60	CS	Yes	15	NED
Heller et al [12]	2014	37	CS	NA	5	Recurrence
Dobrosz et al [32]	2014	42	CS	Yes		NED
Liu et al [33]	2014	39	CS; scar endometriotic nodule excision.	Yes	10	DOD
Aust et al [34]	2015	47	CS	No	10	NED
Sosa- Duran et al [35]	2015	45	CS	Yes	16	NED
Ferrandina et al [2]	2016	44	CS	Yes	6	DOD
Wei & Huang [10]	2017	46	CS	Yes	3	NED
Marques [36]	2017	47	CS	Yes	36	NED
Current case	2018	48	CS; scar endometriotic nodule excision; LH + BSO for endometriosis.	No	2	NA

CS, cesarean section; TAH, total abdominal hysterectomy; BSO, bilateral salpingo-oophorectomy; LH, laparoscopic hysterectomy;

NED, no evidence of disease; DOD, died of disease; NA, not available.

## Data Availability

Our conclusions arise from the evaluation of the histopathologic findings described in this study. No other data can be released due to patient confidentiality.
